# Magnetic Resonance Imaging Volumetry of Primary Nasopharyngeal Cancer in Patients Treated with Induction Gemcitabine and Cisplatin Followed by Concurrent Cisplatin and Volumetric Modulated Arc Therapy

**DOI:** 10.7759/cureus.3296

**Published:** 2018-09-13

**Authors:** Joshua Giambattista, Nevin McVicar, Sarah Hamilton, Montgomery Martin, Benjamin Maas, Cheryl Ho, Jonn Wu, Eric Tran, John Hay, Eric Berthelet

**Affiliations:** 1 Radiation Oncology, British Columbia Cancer, Vancouver Cancer Centre, Vancouver, CAN; 2 Medical Physics, British Columbia Cancer, Vancouver Cancer Centre, Vancouver, CAN; 3 Radiology, British Columbia Cancer, Vancouver Cancer Centre, Vancouver, CAN; 4 Medical Oncology, British Columbia Cancer, Vancouver Cancer Centre, Vancouver, CAN

**Keywords:** nasopharyngeal carcinoma, induction chemotherapy, concurrent chemoradiation, magnetic resonance imagining (mri)

## Abstract

Introduction

The addition of induction chemotherapy (IC) to the standard concurrent chemoradiotherapy (CCRT) is under consideration in locally advanced nasopharyngeal carcinoma (LANPC). To-date, no studies have reported primary gross tumour volume (GTVp) changes using gemcitabine and cisplatin as the IC phase in LANPC. We investigated the timing and magnitude of GTVp response throughout sequential gemcitabine and cisplatin IC and CCRT for LANPC. Toxicity and tumour control probability (TCP) analyses are also presented

Methods

Ten patients with LANPC underwent sequential IC and CCRT between 2011 and 2015. All patients had magnetic resonance imaging (MRI) at three time points: before IC (MRI_0_), after IC (MRI_1_), and three months after CCRT (MRI_3_). Five of the 10 patients had an additional MRI four to five weeks into CCRT (MRI_2_). GTVp contours were delineated retrospectively using contrast-enhanced MRIs, and each GTVp underwent secondary review by a neuroradiologist. Acute toxicities were graded retrospectively via chart review based on the National Cancer Institute Common Terminology for Adverse Events version 4.0 (NCI CTCAE v4.0).

Results

Mean GTVp reduction between MRI_0 _- MRI_1_ was from 68 cc to 47 cc and from 47 cc to 9 cc between MRI_1 _- MRI_3_. In patients with MRI_2_, the mean GTVp reduction between MRI_1 _- MRI_2_ was from 57 cc to 32 cc. Tumour control probability estimates increased by 0.11 after IC. Patients tolerated the treatment well with one Grade IV toxicity event.

Conclusion

The observed GTVp response and improved tumor control probability support further investigation into the use of IC in LANPC.

## Introduction

Locally advanced nasopharyngeal carcinoma (LANPC) is defined by the invasion of a primary gross tumour into adjacent anatomy, including the skull base and/or paranasal sinuses (T3) or intracranial extension and/or involvement of the cranial nerves, hypopharynx, orbit, or extension to infratemporal fossa (T4) [1-2]. The current standard of care for LANPC is cisplatin-based concurrent chemoradiotherapy (CCRT) [3-6]; however, the feasibility of delivering radical radiotherapy may be complicated by the anatomical proximity of critical organs at risk (OARs) [7].

In the Meta-Analysis of Chemotherapy in Nasopharynx Carcinoma (MAC-NPC), which included 19 trials and over 4,800 patients, CCRT had a demonstrable improvement in survival. However, induction chemotherapy (IC) demonstrated no survival advantage (hazard ratio 0.96, 95% confidence interval (CI) 0.80 - 1.16) [8]. A recent Phase III trial demonstrated improved failure-free survival (80 vs 72%, p = 0.034), overall survival (92 vs 86%, p = 0.029), and distant failure-free survival (90 vs 83%, p = 0.031) at three year follow-up in patients with LANPC receiving cisplatin, fluorouracil, and docetaxel IC and CCRT compared to patients receiving CCRT alone [9]. This trial, plus three ongoing Phase III trials, will help to define any survival benefit of sequential IC, plus CCRT, over CCRT alone in LANPC [10-12].

Based on the limited evidence to support the use of IC, the rationale must be carefully considered when developing a treatment plan. Based on the difficult anatomical location of disease, sequential IC may be used to reduce primary gross tumour volume (GTVp) bulk prior to delivering CCRT [3, 5]. There is a paucity of reports on the efficacy of sequential IC and CCRT for reducing GTVp bulk in LANPC. To date, no studies have reported GTVp changes using gemcitabine and cisplatin as an IC phase in LANPC. The primary aim of this work is to describe GTVp changes after IC and assess the potential impact of tumour response on subsequent CCRT. In addition, we follow GTVp during CCRT and three months post-treatment in LANPC using magnetic resonance imaging (MRI) volumetry. Toxicity and tumour control probability (TCP) analyses are also presented 

## Materials and methods

Criteria for eligibility

Between February 2011 and March 2015, 174 cases of NPC were referred to our institution. Of these, 149 patients were treated, including 98 patients that received CCRT. Of the 98 patients receiving CCRT, 37 also had IC. Eligibility criteria for this study included: T3 or T4 primary lesion (i.e., LANPC), delivery of two to three cycles of IC prior to CCRT, and availability of at least three MRIs, including an MRI prior to IC (MRI_0_), following IC and before CCRT (MRI_1_), and three months after completion of CCRT (MRI_3_). In total, 10 patients were eligible and included in this study. Five of 10 patients had an additional MRI four to five weeks into CCRT (MRI_2_). This study was approved by the University of British Columbia, BC Cancer Agency Research Ethics Board (approval #H14-01270).

IC and CCRT

IC consisted of cisplatin and gemcitabine given every 21 days for two to three cycles. Cisplatin was administered intravenously 80 mg/m^2^ on day 1 of each cycle and gemcitabine 1,250 mg/m^2^ was given intravenously on days 1 and 8. During CCRT, cisplatin was given weekly at a dose of 40 mg/m^2^ concurrently with radiotherapy. The selection of cisplatin and gemcitabine as an induction regimen was based on Phase II data [13-16] and the efficacy in the metastatic setting [17]. The evidence suggested a high response rate which was critical for the goal of reducing tumor volume to facilitate radiotherapy delivery.

Pre-IC GTVs were treated with 70 Gy in 35 fractions using volumetric modulated arc therapy (VMAT). If treatment to the pre-IC GTVs was not achievable due to the dose to organs at risk (OARs) exceeding Quantitative Analyses of Normal Tissue Effects in the Clinic (QUANTEC) tolerances, then post-IC GTVs were treated up to 70 Gy, and the pre-IC GTVs received 56 - 70 Gy.

Toxicity

Acute toxicities were graded retrospectively via chart review based on the National Cancer Institute Common Terminology for Adverse Events version 4.0 (NCI CTCAE v4.0) [18]. Rates of Grade III/IV toxicity reported from the time of IC to the three-month follow-up MRI were included. The incidence of a gastrostomy tube (G-tube) placement for symptomatic mucositis and/or weight loss > 10% was also recorded.

GTVp delineation

GTVp included the primary tumour, plus involved retropharyngeal lymph nodes visible on diagnostic MRI. GTVp contours were delineated retrospectively using fat-suppressed, gadolinium-enhanced T1-weighted MRIs by one primary observer and reviewed by a second radiation oncologist. Each GTVp underwent a secondary review by a neuroradiologist. GTVp contours are labeled according to the MRI time point. For example, GTVp_0_ is delineated on MRI_0_; GTVp_1_ is delineated on MRI_1_, and so on. Contouring was performed using the ARIA™ contouring platform (Varian Medical Systems, Palo Alto, CA).

GTVp response

Absolute volumes of each GTVp were calculated in ARIA. The percent volume tumour response (PVTR) between phases *i* and *j*, denoted as PVTR_i-j_ was calculated using equation (1).

$\textrm{PVTR}_{i-j}=100\left ( \textrm{GTVp}_{i}-\textrm{GTVp}_{j} \right )/\left ( \textrm{GTVp}_{0} \right )$ (1)

TCP calculation

TCP was calculated using equation (2) as described by Lee et al. [14-16].

$\textrm{TCP}=K\int_{-\infty }^{\infty }e^{\left ( -\frac{\left ( \alpha -\alpha _{0} \right )^{2}}{2\sigma ^{2}} \right )}T\left ( \alpha \right ) d\alpha$ (2)

where α_0_ is the mean value of α and *K* is the normalization factor for the Gaussian distribution of α. To be consistent with Lee et al., the values α_0_ = 0.31 Gy^-1^, σ = 0.06 Gy^-1^ were used. T(α) represents the TCP for each value of α.

$\textrm{T}=e^{\left ( -\rho \sum V_{i}e^{\left ( -\alpha D_{i}-\beta \frac{D_{i}^{2}}{n} \right)} \right )}$ (3)

where *V*_i_ is the volume of GTVp in cc receiving a total dose of *D*_i_ over n fractions and ρ is the clonogenic cell density. Again, to be consistent with Lee et al., ρ = 10^7^ per cc and α/β = 10 were used.

The paired sample t-test was used to compare changes in volume and TCP pre- and post-IC.

## Results

Patient characteristics

Patient characteristics are described in Table 1. All patients were Epstein-Barr virus in-situ hybridization positive and had non-keratinizing undifferentiated (World Health Organization (WHO) Type III) nasopharyngeal carcinoma (NPC) on pre-treatment biopsy.

**Table 1:** Patient characteristics ECOG: Eastern Cooperative Oncology Group; T-Stage: tumor stage

Gender	
Male	8
Female	2
Age (years)	
Mean	45
Range	33 - 57
ECOG Performance	
0	8
1	2
2	0
T-Stage	
T3	4
T4	6

Toxicity

All patients successfully completed IC and CCRT. Overall, three of 10 patients had Grade III acute toxicity throughout treatment (Table 2). One of 10 required G-tube feeding, and no patients required hospital admission throughout treatment. 

**Table 2:** Summary of grade III acute toxicities G-tube: gastrostomy tube

Grade III Acute Toxicity	
Induction Chemotherapy		
Neutropenia	2	
Anemia	2	
Thrombocytopenia	1	
Vomiting	1	
Concurrent Chemoradiotherapy		
Mucositis at irradiated site	2	
Dermatitis	3	
Nausea	1	
Neutropenia	1	
Anemia	1	
Neuropathy	1	
G-tube insertion	1	

GTVp response to sequential IC and CCRT

**Figure 1:**
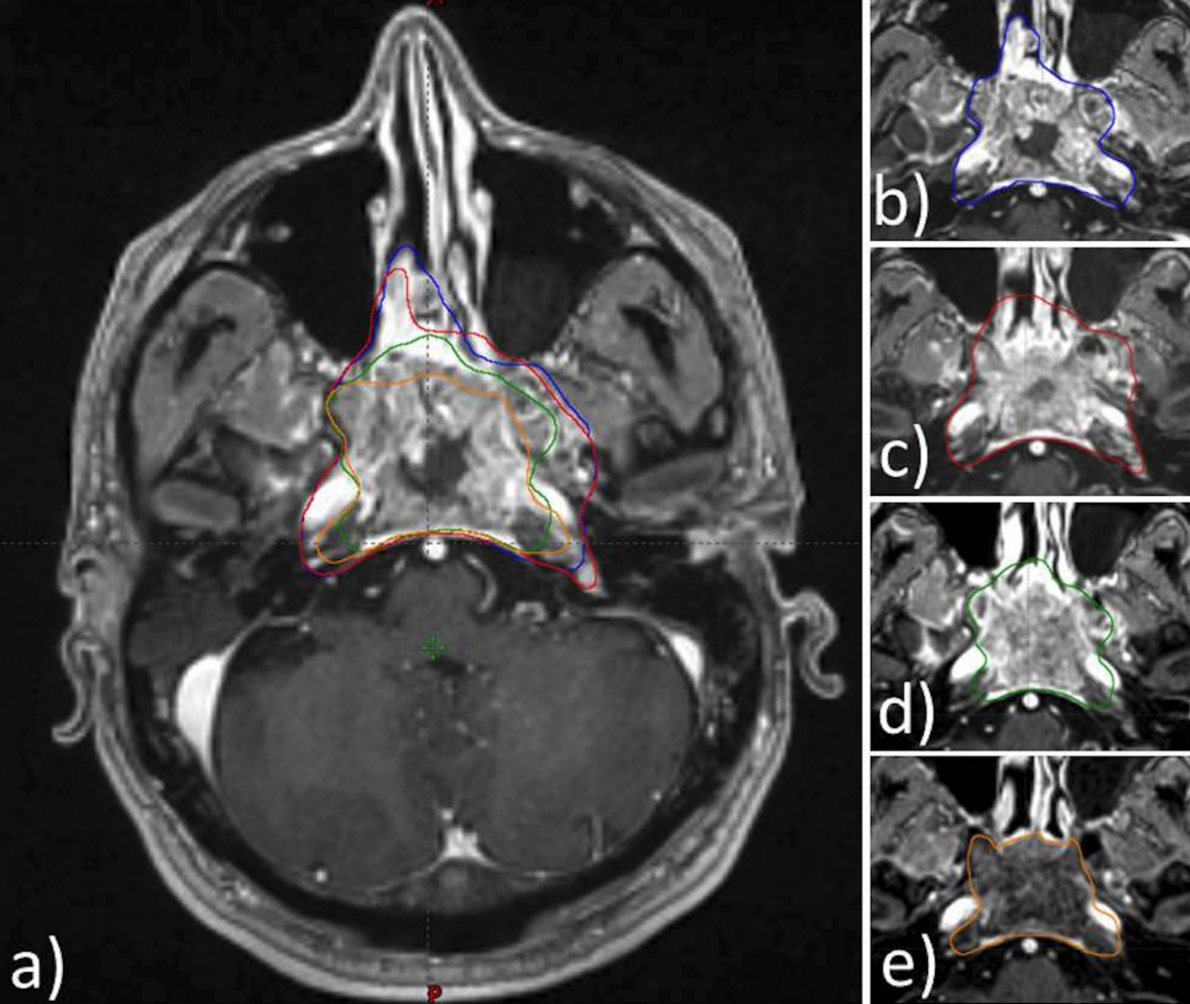
A representative set of axial MRIs acquired for Patient 2 that clearly demonstrates GTV reduction at each time point. a) All time points; b) GTVp_0_; c) GTVp_1_; d) GTVp_2_; e) GTVp_3_ MRI: magnetic resonance imaging; GTV: gross tumor volume; GTVp: primary gross tumor volume

Figure 1 illustrates a representative set of axial MRIs acquired for Patient 2 that clearly demonstrates GTV reduction at each time point. 

**Figure 2:**
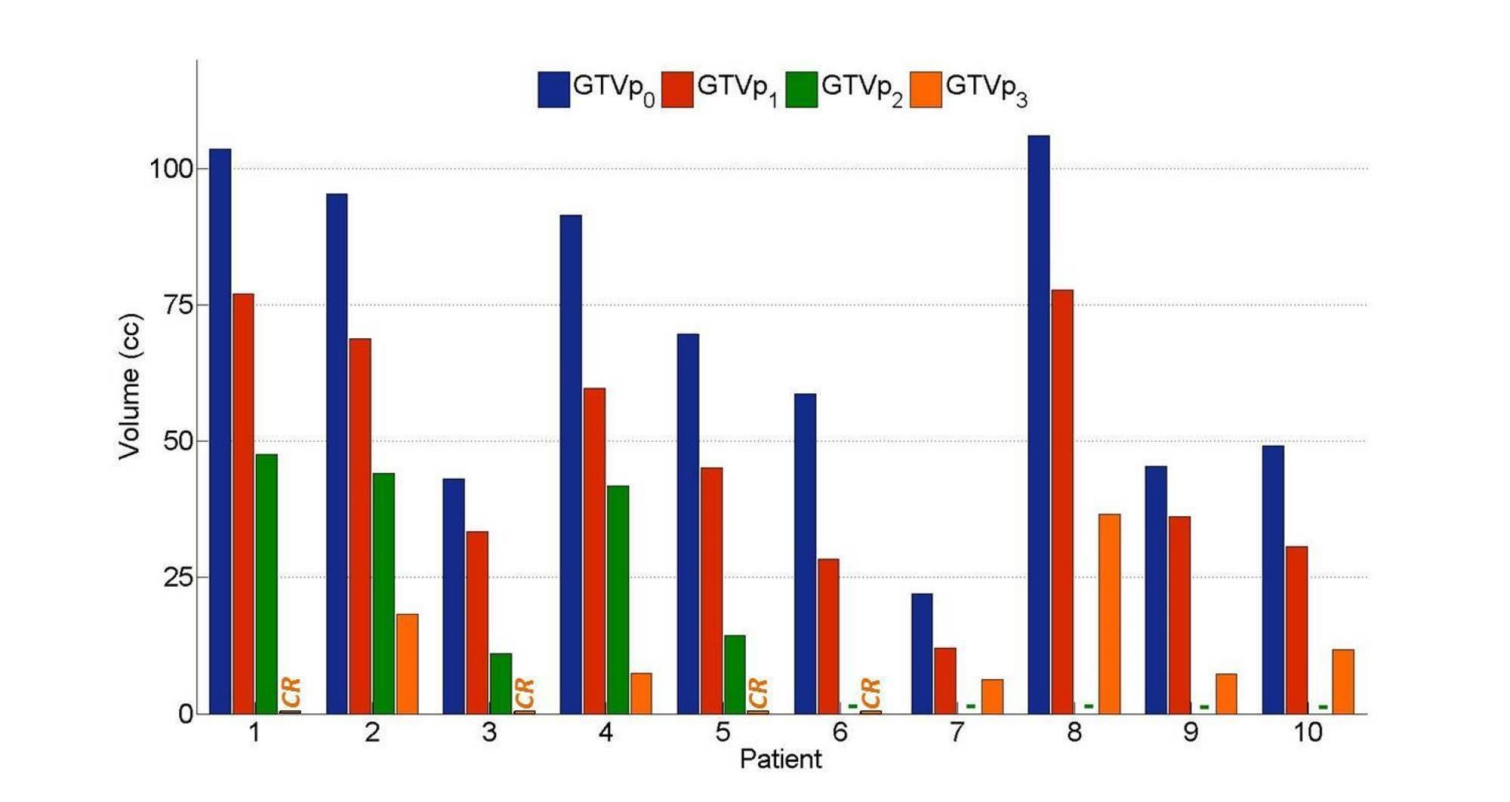
Volumetric response to sequential IC and CCRT Primary gross tumour volumes (GTVp) measured in cc using gadolinium-enhanced MRIs acquired before treatment (GTVp_0_); after induction chemotherapy (GTVp_1_); after four to five weeks of CCRT (GTVp_2_); and at three-month follow-ups (GTVp_3_). Patients 1, 3, 5, and 6 had complete responses at three-month follow-ups as indicated by the ‘CR’ label. Patients 6-10 did not have MRIs after four to five weeks of CCRT as indicated by ‘-’ label. IC: induction chemotherapy; CCRT: concurrent chemoradiotherapy; MRI: magnetic resonance imaging

Volumetric response is summarized for each patient throughout treatment in Figure 2. Four of 10 patients had a complete response, and nine of 10 of patients exhibited > 70% GTVp reduction at three months follow-up. GTVp measured in cc after each treatment phase (GTVp_#_: mean, median, range) were as follows: GTVp_0_: 68, 64, 22-106; GTVp_1_: 47, 41, 12-78; GTVp_2_: 32, 42, 11-48; and GTVp_3_: 9, 7, 0-36. 

**Figure 3:**
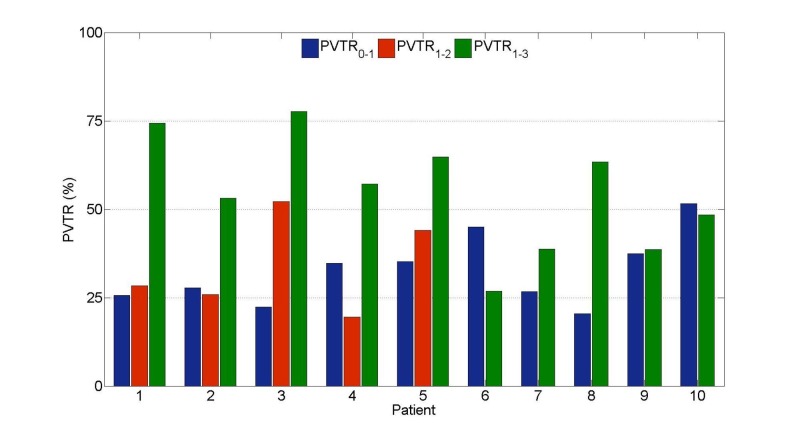
Percent volume tumour reduction (PVTR) from baseline during induction chemotherapy (IC) and concurrent chemoradiotherapy (CCRT) PVTRs calculated using equation (1) and GTVp data shown in Figure 1. Significant (> 20%) tumour response throughout IC (PVTR_0-1_) and throughout the initial four to five weeks of CCRT (PVTR_1-2_) was consistently observed. GTVp; primary gross tumor volume

PVTR throughout each phase of treatment is highlighted in Figure 3. All patients had significant GTVp reduction over the entire treatment, with a mean PVTR_0-3_ of 86% (range: 65 - 100%). During IC, the mean PVTR_0-1_ was 31% (range: 20 - 52%) and for CCRT, the mean PVTR_1-3_ was 51% (range: 27 - 68%). Between the completion of IC and three months post-treatment (PVTR_1-3_), more than a 50% reduction in GTVp was observed in six of 10 patients. In the subset of the five patients who underwent an MRI_2_ after four to five weeks of CCRT, the mean PVTR_1-2_ was 32% (range: 20 - 52%).

Impact of IC on TCP and volume

IC decreased the volume in all patients (Table 3). Prior to IC, the mean volume was 68.5 cc (median 64.5, range: 22 - 106) and after IC, the mean volume was 46.9 cc (median: 40.5, range: 12 - 78), p < 0.0001. IC improved TCP in all patients. Prior to IC, the mean TCP was 0.65 (median: 0.72, range: 0.14 - 0.89), and after IC, the mean TCP increased to 0.76 (median: 0.84, range: 0.42 - 0.90), p = 0.005. Patient 1 had the lowest pre-IC TCP value and exhibited the largest improvement in TCP (+0.28) following IC. Three patients had pre-IC TCP values > 0.80 and each exhibited small TCP improvements of +0.01. 

**Table 3:** Summary of GTVp and TCP changes due to IC GTVp: primary gross tumour volume; TCP: tumour control probability; IC: induction chemotherapy; Δ: delta

Patient	Volume (cc)			TCP		
	Pre-IC	Post-IC	$\Delta$	Pre-IC	Post-IC	$\Delta$
1	104	77	27	0.14	0.42	+0.28
2	95	69	26	0.82	0.83	+0.01
3	43	33	10	0.87	0.88	+0.01
4	92	60	32	0.69	0.81	+0.12
5	70	45	25	0.75	0.85	+0.10
6	59	28	31	0.61	0.69	+0.08
7	22	12	10	0.89	0.90	+0.01
8	106	78	28	0.64	0.85	+0.11
9	45	36	9	0.30	0.48	+0.18
10	49	31	18	0.79	0.86	+0.07

## Discussion

To our knowledge, this is the first report on GTVp response to IC using gemcitabine and cisplatin in LANPC using MRI. We included involved retropharyngeal nodes due to the fact that they were often contiguous with the GTVp.

Overall, patients demonstrated good tolerance to gemcitabine and cisplatin IC. Other groups have reported excellent compliance and similar toxicity in patients receiving various IC regimes prior to CCRT [9, 14, 17-21]. Hui et al. found similar toxicity profiles in patients receiving docetaxel and cisplatin-based IC regimes, except for a high occurrence (97%) of Grade III/IV neutropenia during IC [22]. Fountzilas et al. reported acceptable patient tolerance to IC consisting of epirubicin, paclitaxel, and cisplatin, followed by cisplatin-based CCRT, although the rate of thrombocytopenia was higher with IC [23]. Recently, Yang et al. demonstrated acceptable toxicities in LANPC patients receiving IC regimens consisting of paclitaxel with cisplatin or 5-fluorouracil with cisplatin [24].

It is widely accepted that most LANPC tumours shrink in response to IC regimens; however, there is limited data on the timing and magnitude of IC contributions to tumour response. Accurate volumetry is necessary in order to optimize the potential therapeutic benefits of sequential IC and CCRT. In radiotherapy, therapeutic benefit improves by increasing the dosimetric coverage of the clinical target volume and/or decreasing dose received by critical OARS. Hence, the two general strategies to exploit GTVp response during IC include 1) treating the smaller post-IC GTVp, thereby improving the sparing of nearby OARS, or 2) treating the larger pre-IC GTVp, thereby improving the dosimetric coverage of the post-IC CTV. Lee et al. judiciously reported that treatment of the larger pre-IC GTVp is more prudent until further study characterizing the post-IC tumour extent [19]. More recently, a randomized clinical trial reported that treating post-IC GTV led to improved quality of life without reducing local control and survival in LANPC patients [25].

Lee et al. showed that IC with cisplatin and 5-fluorouracil in LANPC patients led, on average, to a 61% reduction in GTVp using MRI volumetry [19]. They calculated that the tumour response to IC improved TCP from 0.83 (pre-IC) to 0.89 (post-IC). The 33% GTVp reduction during IC observed in our study is considerably lower; however, differences are expected due to differing IC regimens, GTVp definitions, and patient populations. We calculated a similar improvement in TCP of +0.11. In the current study, TCP values are consistently lower than those reported by Lee et al. and this may reflect differences in aggressiveness of treatment and/or patient selection. For TCP calculation, we used the identical biological parameters to guide comparisons. Differences in the patient population will influence the accuracy of TCP values reported in our study.

TCP is a metric traditionally used to compare different radiotherapy regimens in terms of their respective probability of achieving tumour eradication. TCP values must be viewed as relative estimates since their calculation is based upon simplified statistical models of malignant clonogenic cells response to irradiation. Furthermore, TCP values do not account for the tumoricidal effects of chemotherapy.

Adaptive re-planning has been proposed in LANPC for patients with GTV reduction throughout treatment. Re-planning partway through treatment using smaller GTVs can enable a more curative dose to be delivered to the GTV while adhering to strict dose limitations to nearby neurologic structures often compromise radiotherapy plans [8, 26-27]. Chen et al. reported improved two-year local control of 88% versus 79% for appropriately selected patients treated with adaptive re-planning compared to patients treated without re-planning [28]. Patients selected for re-planning had worse disease and tumour stage compared to patients who did not, yet they demonstrated a higher rate of local control. Several studies reported that the ideal time point for adaptive re-planning is four to five weeks into CCRT due to OAR movement and GTVp shrinkage [27, 29]. In our study, the subset of patients with MRI_2_ demonstrated an average of 65.2% reduction of GTVp_0_ by this time point. Future studies are needed to investigate whether PVTR_0-2_ is different between patients receiving sequential IC and CCRT versus CCRT alone.

The temporal pattern of GTVp response varied among this small group of patients. For example, Patient 3 had a 22% reduction in GTVp during IC, whereas Patient 6 had a 51% reduction. Interestingly, both achieved complete response by MRI_3_. The observed response to IC did not correlate with tumour response to CCRT in our small sample of patients. Sze et al. found that large pre-treatment GTVp corresponded to a worse prognosis [30]. In our study, Patients 1 and 8 had the largest GTVp_0_ and both had similar responses to IC (26% and 27% reduction, respectively). However, Patient 1 went on to have a complete response, while Patient 8 had a poor overall response with significant residual GTVp_3_ at MRI_3_, corresponding to 35% of the initial GTVp_0_.

Inherent limitations of this study include its small sample size and retrospective nature. In addition, three-month follow-ups may not provide sufficient time to observe the total tumour response or potential local recurrent disease since GTVps could continue to change. Our findings reflect a patient population that is characteristic of endemic NPC since all subjects exhibited non-keratinizing pathology; however, we are unable to correlate Epstein-Barr virus (EBV) status with the GTVp response since EBV serology is not routinely obtained at our center [5]. Finally, we do not have a comparable LANPC patient population with similar MRI datasets who did not undergo IC for comparison, as treatment with IC is standard protocol for patients with T3 and T4 disease treated at our center.

The role of IC in a general NPC population is under investigation. Recently completed and ongoing Phase III trials, however, may provide more insight with modern chemotherapy regimens and radiotherapy techniques. It remains that the overall benefit of IC in LANPC is not fully elucidated, and tumor size and anatomic location may provide better guidance for patient selection.

## Conclusions

Our volumetric results support further investigation into the use of IC to reduce GTVp. Larger prospective studies with more frequent MRI evaluation should help to address some of these questions.
